# The Diagnosis of the Internal Secretory Diseases

**Published:** 1917-01

**Authors:** Henry R. Harrower

**Affiliations:** M. D., F. R. C. S. (Lond.), Los Angeles, California


### The Diagnosis of the Internal Secretory Diseases.

BY HENRY R. HARROWER, M. D., F. R. C. S. (LOND.), LOS ANGELES, CALIFORNIA.

### VIII.

#### INTERNAL SECRETORY DISTURBANCES OF THE GONADS.

There are numerous diseases of the gonads, or essential sex glands, which are discussed in any work on surgery or gynecology. The infections and tumors of the testes and ovaries need not be considered here. There are, however, a number of symptoms-complex which result from some derangement of the endocrine functions of the gonads, and to these it is proposed to call attention.

As all know, the ovaries are a far more frequent source of disease, functional or organic, than the testes, though each gland is subject to a number of affections. Let us first devote our attention to the female gonads.

#### PRELIMINARY CONSIDERATIONS.

The biochemical basis of femininity is more important than either the psychological basis, or that dependent upon the nervous system. The homones transcend in importance all other factors in the regulation of the chemistry of the body and, therefore, the chemistry of the reproductive organs, hence derangement in the glands of internal secretion, particularly those under discussion for the moment, spells deranged metabolism, disturbed nutrition and altered sex conditions.

The whole subject is handled in a masterly and most interesting fashion in the recently published book, "The Sex Complex,"*

*"The Sex Complex: A Study of the Relationships of the Internal Secretions to the Female Characteristics and Functions in Health and Disease," by W. Blair Bell, B. S., M. D., Lond., Gynecological Surgeon by my friend, Dr. W. Blair Bell, of Liverpool, from which a few short quotations will be made later. The least that I can say about this book is that it is of fascinating interest and much practical value to the reader.

For convenience, the experimental evidence which has established the almost universal belief that the ovaries produce one or more internal secretions, must be omitted, though it may be stated that the ovary presides over genital development and in a large measure is concerned in bringing about the changes in feature, form and function which accompany the commencement and cessation of sexual life—puberty and the menopause—as well as other well known changes in the interim.

From the standpoint of endocrinology, and especially the diagnostic aspect of it, the ovaries are subject to three form of functional disorder: (1) Deficient secretion; (2) excessive secretion and (3) perverted secretion. The results of these will be considered briefly and the essentials of their diagnosis outlined here.

### OVARIAN INSUFFICIENCY.

Hypo-ovarism or ovarian insufficiency may be spontaneous in origin or it may be an acquired condition; that is to say, the change may be initiated sufficiently early to prevent the normal development and growth dependent thereon; or, on the other hand, disease of the ovaries may supervene after ovarian function or maturity has been fully established with certain fairly well-defined results.

The former rendition naturally implies a wider and more fundamental symptomatology, for the changes of puberty are purely of endocrine origin and the ovaries are among the principal agencies in bringing them about.* The results of early ovarian insufficiency are combined and generally known as "infantilism." The usual findings include delayed or arrested growth of the body as a whole and of the reproductive organs in particular. The

---

to the Royal Infirmary, Liverpool; Hunterian Professor in the Royal College of Surgeons, England, etc. New York: William Wood & Son, 1916. Pp. 233.

*Though it is well established that the thyroid, thymus, pituitary and adrenal cortex, and possibly others of the ductless glands including the pineal body, exert a controlling influence upon the function of the ovaries and that disturbance of these glands very commonly are reflected in the physiological activities connected with ovarian function. For this reason mistakes have been made in charging to the gonads what is really of more remote origin. Incidentally the aphorism, "Monoglandular disease is rare; pluriglandular disorder is the rule," will bear careful remembrance when studying the aspects of ovarian disease.

breasts may be small and undeveloped, though not infrequently this does not appear to be the case, especially when there is plenty of fat in the tissues. The hips are narrow. The pubic and axillary hair is scanty or absent; and the psychic and sensory evidences of sex are diminished in degree or entirely absent.

The later onset of ovarian insufficiency is not accompanied by such well-marked evidences, at least as far as physical development is concerned, for obvious reasons; but the functional changes are usually clearly discernible. Since the growth of the myometrium and, in fact, the pelvic circulation and reproductive development are under hormone control, in cases of ovarian insufficiency the uterus may be expected to be infantile (or "senile") and the adnexa undeveloped or atrophied.

Where this is acquired in later life, genital atrophy is to be seen as a shrinking of the internal and external genitalia.* The labia majora diminish in size, while the labia minora are slender and insignificant and may disappear entirely. The introitus narrows and tends to become wave-like, and the modified vaginal membrane is thin, pale and mottled, later becoming tough and unyielding. The vagina contracts and obstructing bands may be formed, while the cervix shrinks and its lumen tends to close.

Quite the most common clinical evidence of hypo-ovarism is amenorrhea; and this symptom may be seen in all grades from complete absence to bimonthly or more widely separated "occasional" periods, and from regular molimina with practically no flow (and, often, a good deal of pain and physical discomfort) to irregular and widely separated periods with a seemingly normal amount of menstrual discharge and little or no physical discomfort with it.

The general health may be quite good, and in young girls no special attention be paid to the asexual condition until several years after the normal time to mature. On the other hand, malnutrition and, particularly, anemia are usual accompaniments of ovarian insufficiency.

Reflex troubles are often found, the most common among which is headache. Hysteria has been noted many times and "neurasthenia" is often the label improperly attached to hypo-ovarism or, more properly, pluriglandular insufficiency with ovarian involvement.

---

*See W. P. Graves, "Practical Aspects of the Ovarian Secretions," *New York State Journal of Medicine,* August, 1916.

With the above changes, one would naturally expect to find sterility, and this is the rule (though there are exceptions to it). Indeed, sterility may be the only sign of ovarian insufficiency. Repeated early abortions have been seen by the writer in which the cause evidently was not so much defective ovulation or impregnation (syphilis having been ruled out) as a difficulty with the proper implantation of an apparently normal embryo, a function which is now conceded to be made possible through hormone influences, and which, by the way, occasionally may be remedied by suitable organotherapy.

There is an aspect of diminished ovarian secretion in relation to the mentality which may be mentioned. This is the hypo-ovarism occasionally accompanied by evidences of masculinity or, at least, reduced femininity. This is interestingly referred to by Blair Bell, from whose book (page 207) the following is quoted: "When any of the masculinity-producing internal secretions become abnormally active in women the ovarian secretions are antagonized or inhibited, partially or completely, and the metabolism is directed towards the necessities of masculinity. In such circumstances, with the development of physical changes towards masculinity, the mind becomes less feminine in its outlook. Cases of so-called 'true hermaphroditism' are rare; but there is little doubt that women with a larger share of masculinity than is normal are extremely common. These unnatural individuals are easily detected by the coarseness of their skins, by the size of their extremities, by the ill-development of their breasts and by the assertiveness and aggressiveness of their conversation and schemes. No doubt many of them have hyperplasia of the suprarenal cortices or of the pituitary; and the atrophy of the ovaries, when it exists, is secondary to the primary changes in the other organs of internal secretion which are responsible for the development of masculinity."

### THE MENOPAUSE.

Strictly speaking, the "change of life" is a physiological hypo-ovarism—the removal of the chemical factor in the hormone balance to which the organism has accustomed itself for thirty or more years. The organic changes are too well known to require reiteration here and include most of the retrograde changes previously mentioned. The functional derangements accompanying the climacteric are very common, and are a subject of special interest, since they often constitute a form of hypo-ovarism which responds to ovarian therapy and, too, they are not infrequently brought about prematurely by necessary or unnecessary surgical interference.

The circulatory, sensory and psychic aberrations of climacteric origin include irregular uterine hemorrhage, "flashes of heat," pelvic fullness due to congestion, headaches, fleeting and indefinite pains and sensory disturbances in various localities, irritability, melancholia or depression, and the well known but little understood "neurasthenia." Vaso-motor derangements following castration or the menopause may also follow functional ovarian insufficiency due to local disease. These functional disorders of the menopause usually may be treated as hypo-ovarism with encouraging results.

### HYPEROVARISM.

Excessive internal secretory activity on the part of the ovaries or hyperovarism is not so frequently encountered as ovarian insufficiency. Rarely in early life it may accompany pituitary disease, abnormal thymus atrophy or a pineal tumor and, as a result of the disturbed hormone stimuli, the ovaries may commence to functionate very early. Cases are on record where the evidences of puberty were present at five or six years and from a reproductive standpoint, while procreation may not have been possible, at least such cases were rightly classed as "precocious."

For reasons that are not always clear—psychic, functional or organic—the ovaries may produce an undue amount of their internal secretion. The resultant symptoms include menorrhagia (frequent menstrual periods or an excessive flow at proper intervals), sexual or mental supersensitiveness and pelvic pain and a sense of uncomfortable fullness in the lower abdomen. The external genitalia are irritable and the condition known as nymphomania may be purely of ovarian origin. Hysteria also may be directly due to this disturbance.

The adrenal glands may be excessively stimulated by this abnormal secretory action of the ovaries, which accounts for the ease with which such individuals become fatigued and the ultimate asthenia or adynamia so common in certain "ovarian cases." Here, however, it should be remembered that numerous other circumstances may be the cause of adrenal depletion and the consequent asthenia while accompanying other evidences of hyperovarism may really be due to other remote causes.

Cases of functional hyperovarism usually exhibit sexual neuroses, and these results may include masturbation and nymphomania, growing into "sexual insanity."

There is a therapeutic-diagnostic test which is well worth trying in hyperovarism: Functional menorrhagia and other conditions purely due to ovarian excess (not to new growths or to mechanical causes) are often modified by mammary organotherapy. Five to ten grains of desiccated mammary gland given three times a day before meals have controlled the hemorrhage and pelvic uncomfortableness very nicely. At the same time this assists in establishing the functional basis of the disorder. (Parenthetically the internal secretion of the mammary glands exerts an antagonistic action over that of the ovaries, as does that of the pancreas over the secretion of the adrenal medulla, and vice versa.)

One of the chemical results of hyperovarism is especially noticeable in osteomalacia. This lack of lime and softening of the bones is now known to be intimately connected with the glands of internal secretion and particularly the ovaries. Osteomalacia may be brought about directly by ovarian excess (and be remedied very largely by removal of a portion of the hyperactive glands, just as the thyroid is removed, in part, in hyperthyroidism, etc.). In these cases the disordered calcium metabolism is due probably to the abnormal excretion of lime brought about by the undue ovarian stimuli.* This condition is not usual in nonpregnant women, as they do not have the great need for lime that is present during pregnancy; but since child-bearing causes a large demand for extra lime, softening of the bones may occur and is not uncommon in Italy, Austria and India. At one time osteomalacia was routinely treated by oophorectomy, but since Bossi, in 1907, first suggested the administration of an antagonizing hormone instead of ovarian removal, adrenal substance has been given with many resulting cures. More recently Blair Bell has directed a series of cases in India, at long distance, and the posterior pituitary principle has been given in osteomalacia with distinct benefit in a number of cases. This seems to indicate that osteomalacia is likely a pluriglandular disorder, the hyperovarism being coupled with hypoadrenia or hypopituitarism. This is undoubtedly the case, and indicates, at least, a prospective line of

*Blair Bell has shown by numerous experiments that the ovaries are an important factor in the regulation of the power of the organism to appropriate calcium; and the clinical experiences with osteomalacia seem to prove his contention.

treatment of hyperovarism where ordinary treatment is unavailing and operation inadvisable.

DYSOVARISM.

The third form of ovarian dysfunction is not unusual and is a form of gonad dyshormonism that really results from deranged cell activities following new growths or cysts of the ovaries. The frequency of ovarian tumors is responsible for the frequency of dysovarism. This condition is differentiable from hyperovarism and the clinical findings are irregular since hyperovarism may be accompanied by periods of ovarian excess or insufficiency.

Occasionally there is produced in the ovarian tissue (either in the normal interstitial or luteal cells, or in those of the new growth) a toxic hormone of extreme virulence and in comparatively recent German literature the term "ovarian poisoning" is found, denoting a vicious activity of diseased ovarian tissue with serious remote effects due to the poisons produced there and secreted directly into the blood stream as are practically all the hormone bearing internal secretions. The treatment, of course, involves the removal of the offending tissue.

Dysovarism may be the cause of alternate periods of amenorrhea and menorrhagia. Dysmenorrhea is the rule. Neurotic manifestations are quite usual, and some have reported insanity as one of the possible results of ovarian derangement. Under certain circumstances an abnormal menopause virtually develops into minor form of dysovarism, the varying symptoms being due to irregular periods of differing ovarian activity.

The most common symptoms of dysovarism are pain in the pelvis and severe asthenia. The extreme prostration and weakness is doubtless due to a superinduced hypoadrenia, and may be the outstanding feature of a case. In most cases on bimanual palpation the offending organ or organs frequently will be found to be nodular, irregular, enlarged and tender. Here again surgery is the proper remedial procedure.

THE MALE GONADS.

Perhaps the dysfunctions of the testes do not require a comprehensive outline, for we are better acquainted with the results of increased or decreased physiological activity of these glands. Hypogonadism virtually means essential infantilism, sexual insufficiency and maldevelopment as cryptorchidism and impotence.

Naturally neurasthenia is common in such cases, and it must be expected that this disorder is by no means limited to the essential sex glands, because of their intimacy with the other glands of internal secretion.

### INFANTILISM.

There are several clinical forms of testicular disorder: Infantilism is the condition in which the glands are poorly developed or absent. Here the results are much the same as in hypo-ovarism, the form and function is changed, the bodily growth is altered and the secondary sexual characteristics which normally should show themselves at puberty do not materialize.

The testicles are very small, the scrotum atrophied and the penis short and incapable of erection. Infantilism may vary in degree, and developmental changes are not always necessarily accompanied by absolute inactivity of the interstitial cells of Leydig. In this case, there may eventually be possibilities of sexual desire and azoospermia may be absent.

### CRYPTORCHIDISM.

The development anomally known as cryptorchidism is not rare; but cases with permanent cryptorchidism are very uncommon. There may be two forms, the abdominal and the inguinal. Occasionally this condition, also known as "undescended testicle," is accompanied by testicle maldevelopment with lack of all the functions dependent upon proper activity of the Leydig cells. Again, despite complete burial of the testes, they may be functionally active, in which case there is sterility in individuals none the less potent; the sterility being purely mechanical rather than functional. Such cases are not subject to the same degree of asexualism as in pure infantilism or in castrates.

### EUNUCHOIDISM.

Eunuchoidism is presumed to be an acquired disorder of the interstitial cells of Leydig, and those with this disturbance are quite similar in functional incapacity to a castrate but without the absence of the testes. Here there is complete functional loss of the sex principle later in life, so that the more marked manifestations of infantilism are not present.

The eunuchoid is so named from the similarity in form to the eunuch or castrate, and in addition to the retrogressive changes in the secondary sex characteristics—avirilism, reduction of facial, axillary and pubic hair, genital atrophy, there is an acquired corpulency due to the loss of the powerful oxidizing principle produced in the interstitial cells. Eunuchoidism may be due to disease or be a spontaneous hormonically produced disorder, and it is accompanied by a loss of the factors dependent upon active sex-gland function—assertiveness, courage, animation and sexual power. The ergograph has been very effectively used to demonstrate the actual loss of energy and power following disease or injury to the gonads as well as to show the energizing influence of suitable organotherapy or the more recent work by Lydston and others with sex gland transplantation.

### FUNCTIONAL SEXUAL DISTURBANCES.

Many a monograph has been written on this subject, and it is far too large to be considered here. Impotence, presenility and senile testicular insufficiency have always been a subject of perennial interest. From a diagnostic standpoint, the principal symptoms are lack of sexual desire and power and "sexual neurasthenia" with its innumerable manifestations. According to Williams, functional testicular disorders, especially on the side of deficiency, are known to cause general depression, hysteria, hypochondria, melancholia and also digestive disturbances.

It may be well to recall that the fundamental basis of modern organotherapy and the "fillip" which restarted the study of the age-old study of organ medication was the use by Browne-Séquard of testicular extract on himself. The dynamogenic influence of this sort of treatment then, as now, is unquestioned; but for various reasons this form of treatment has never assumed the importance that it really deserves.

Undoubtedly there is such a condition as hypergonadism; but in most cases we have to meet the origin is psychic and usually beyond the control of ordinary medical treatment. From a diagnostic standpoint it is not difficult to determine.

Next month: "The Parathyroids and Pineal."

